# Mix and match in reverse total shoulder arthroplasty: overview of current prostheses systems and potential combinations

**DOI:** 10.1007/s00402-026-06345-w

**Published:** 2026-05-20

**Authors:** Dominik Seiß, Adrian Schlichter, Johannes Maria Graf, Richard Stange, Malte Ohlmeier

**Affiliations:** 1 Department of Trauma-, Hand- and Reconstructive Surgery, University Hospital Münster, Munster, Germany; 2 Department of Trauma-, Hand- and Reconstructive Surgery, University Hospital Muenster Marienhospital, University Hospital Muenster Marienhospital, Munster, Germany; 3 Department of Regenerative Musculoskeletal Medicine, Institute of Musculoskeletal Medicine (IMM), University Hospital Münster, Munster, Germany

**Keywords:** Reverse shoulder arthroplasty, Mix and match, Mismatch implantation, Revision shoulder arthroplasty

## Abstract

**Introduction:**

Mix and Match is considered a viable option for minimizing morbidity in total hip arthroplasty (THA). However in reverse total shoulder arthroplasty (rTSA) this strategy remains comparatively new. This study provides an overview of currently available implant systems and the potential combinations of glenoid and humeral components.

**Materials and methods:**

A review of commercially available shoulder arthroplasty systems was performed. Nineteen manufacturers were contacted and asked to provide detailed information on glenoid and humeral component sizes for their rTSA systems. The reported component sizes were compiled and analyzed for possible combinations.

**Results:**

Thirteen manufacturers (68%) consented to the publication of their component size data. Two manufacturers (11%) do not produce rTSA systems, and four (21%) did not respond to repeated inquiries. A total of 21 implant systems were reported, comprising 10 distinct implant sizes. Glenosphere and humeral inlay diameters ranged from 32 mm to 48 mm, with 36 mm being the most commonly available size (16 of 21 systems, 76%).

**Conclusion:**

Mix-and-match implantation in rTSA is likely employed more frequently than documented, yet remains poorly investigated. This study demonstrates that numerous systems can theoretically be combined during revision surgery, although the biomechanical implications are not yet understood. Early clinical reports appear promising. The potential advantages for patients undergoing this procedure include reduced operative time, less invasive surgery with lower risk of perioperative complications.

**Level of evidence:**

Level V.

## Introduction

The prevalence of reverse shoulder arthroplasty (rTSA) continues to rise [3, 15]. Its indications now extend beyond cuff tear arthropathy to a broader range of shoulder pathologies [4]. In 2017, 63,845 rTSAs were performed in United States [15]. According to Wagner et al. implantation numbers are projected to increase to between 174,810 and 350,558 by 2025 [15]. In the German shoulder prosthesis register, 5491 primary rTSAs and 286 revision rTSAs were recorded between 2006 and 2019 [5]. From 2010 to 2022 implantation numbers in Germany increased from 5326 to 24,067, while revision procedures increased from 2179 to 3893 [1].

According to the DVSE (D-A-CH Vereinigung für Schulter- und Ellenbogenchirurgie)- registry, the leading causes of rTSA revisions between 2006 and 2019 were dislocation (26.2%), periprosthetic joint infection (PJI) (16.1%), humeral stem loosening (12.9%), and glenosphere loosening (12.4%) [5]. Revision surgery often poses significant challenges. Therefore, the least morbid approach is performed, which can necessitate mixing components from different manufacturers.

To date no overview of RSA implant systems including available component sizes and potential mix-and-match combinations has been published. This study addresses this gap.

## Materials and methods

A review of currently available shoulder arthroplasty systems was conducted. Based on an online survey conducted among members of the DVSE regarding lateralization in rTSA by Ohlmeier et al. [7], the manufacturers identified in this survey were contacted to provide detailed information on their currently available rTSA systems, including component sizing, and to obtain consent for publication.

Manufacturers were contacted twice via their publicly available email addresses. Those who did not respond were excluded from the present analysis.

Glenosphere and humeral inlay sizes from consenting manufacturers were compiled.

## Results

Of the 19 manufacturers surveyed, 13 (68%) consented to the publication of their component sizes. Two manufacturers (11%) confirmed that they do not produce RSA systems. Four (21%) did not respond despite multiple written inquiries. No responding manufacturer rejected consent for publication.

**Fig. 1:**
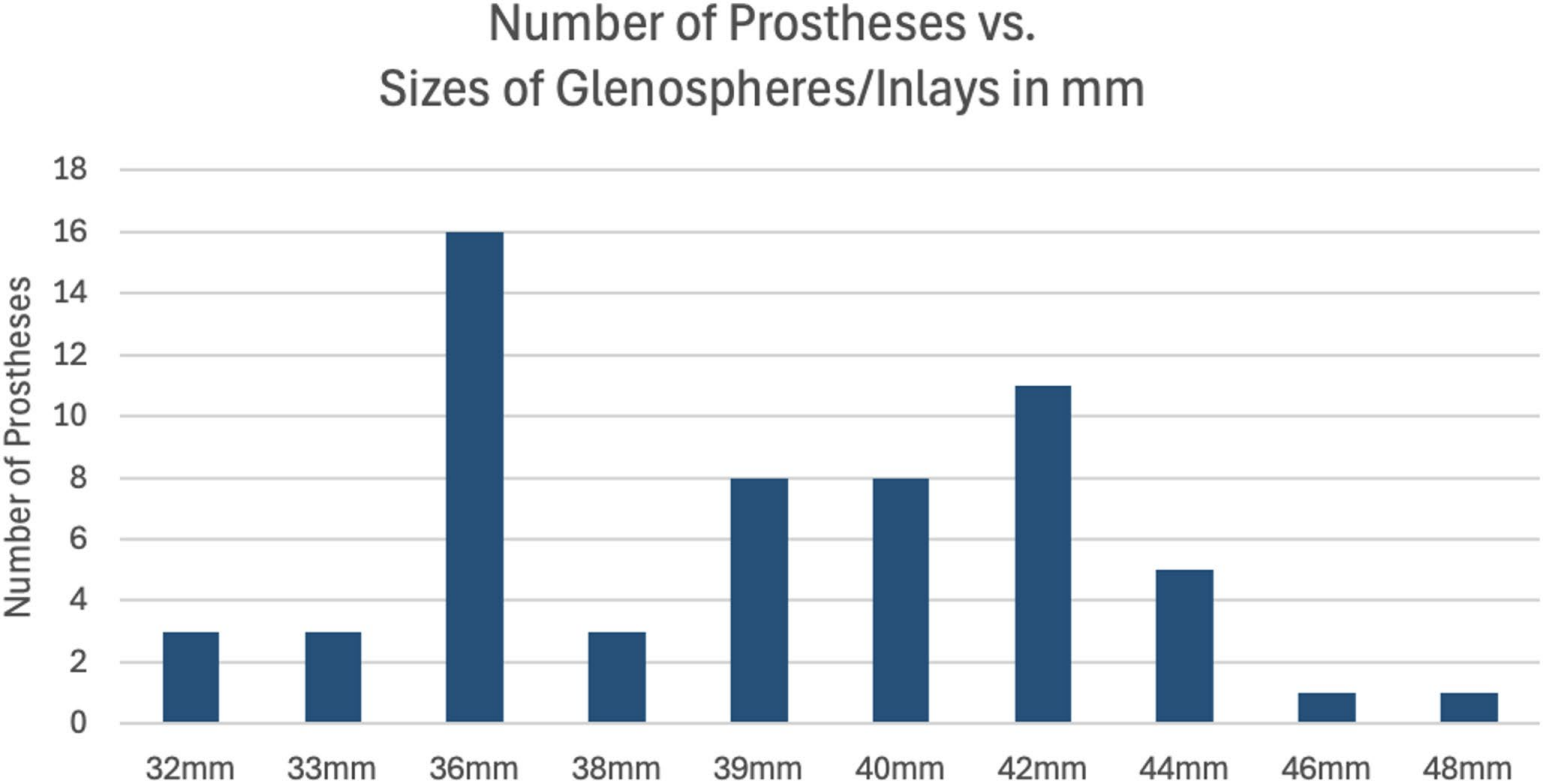
Number of prostheses vs. sizes of glenospheres/Inlays in mm

In total 21 prostheses were reported. Each system included two to four available component sizes, with 10 distinct diameters across all manufacturers. Glenosphere and inlay sizes ranged from 32 mm to 48 mm. The most common size was 36 mm (16 of 21 systems, 76%), followed by 42 mm (11 of 21 prostheses, 52%, see Fig. 1). Sizes 39 mm and 40 mm were each available in eight systems (38%). Size 44 mm is listed in five prostheses systems (24%). Sizes 32 mm, 33 mm and 38 mm are reported in three systems (14%). The least frequent sizes were 46 mm and 48 mm, each appearing in only one system (5%, see Table 1).

**Table 1:** Sizes of glenospheres/Inlays

Manufacturer	Name of prosthesis	Nickel-free prosthesis available?	Size of Glenosphere in mm	32	33	36	38	39	40	42	44	46	48	Size of Inlay in mm	32	33	36	38	39	40	42	44	46	48
Enovis/Mathys	Affinis inverse	Yes		0	0	1	0	1	0	1	0	0	0		0	0	1	0	1	0	1	0	0	0
	Affinis fracture inverse	Yes		0	0	0	0	1	0	1	0	0	0		0	0	0	0	1	0	1	0	0	0
Arthrex	Univers revers MGS	No		0	1	1	0	1	0	1	0	0	0		0	1	1	0	1	0	1	0	0	0
Implantcast	Agilon omarthrosis	Yes		0	0	1	0	0	1	0	1	0	0		0	0	1	0	0	1	0	1	0	0
	Agilon trauma	Yes		0	0	1	0	0	1	0	1	0	0		0	0	1	0	0	1	0	1	0	0
Enovis/Lima Corporate	SMR reverse Mach 1, 2, 3	Yes		0	0	1	0	0	1	0	1	0	0		0	0	1	0	0	1	0	1	0	0
Medacta	Reverse shoulder system	Yes		1	0	1	0	1	0	1	0	0	0		1	0	1	0	1	0	1	0	0	0
Stryker	Tornier perform reversed glenoid	Yes		0	1	1	0	1	0	1	0	0	0		–	–	–	–	–	–	–	–	–	–
	Tornier perform humeral system stem	Yes		–	–	–	–	–	–	–	–	–	–		0	1	1	0	1	0	1	0	0	0
	Tornier aequalis reversed II	Yes		0	1	1	0	1	0	1	0	0	0		–	–	–	–	–	–	–	–	–	–
	Tornier flex	Yes		–	–	–	–	–	–	–	–	–	–		0	1	1	0	1	0	1	0	0	0
	ReUnion RSA	No		1	0	1	0	0	1	0	0	0	0		1	0	1	0	0	1	0	0	0	0
Zimmer Biomet	Comprehensive	Yes		0	0	1	0	0	1	0	0	0	0		0	0	1	0	0	1	0	0	0	0
	Trabecular metal reverse shoulder system	Yes		0	0	1	0	0	1	0	0	0	0		0	0	1	0	0	1	0	0	0	0
Exatech	Equinoxe	No		0	0	1	1	0	0	1	0	1	0		0	0	1	1	0	0	1	0	1	0
Depuy synthes	Global unite	No		0	0	0	1	0	0	1	0	0	0		0	0	0	1	0	0	1	0	0	0
	Delta XTEND	No		0	0	0	1	0	0	1	0	0	0		0	0	0	1	0	0	1	0	0	0
Link	Embrace	Yes		0	0	1	0	1	0	1	0	0	0		0	0	1	0	1	0	1	0	0	0
Enovis	AltiVate reverse	No		1	0	1	0	0	1	0	1	0	0		1	0	1	0	0	1	0	1	0	0
FH Orthopedics	Arrow universal shoulder prosthesis	No		0	0	1	0	1	0	1	0	0	0		0	0	1	0	1	0	1	0	0	0
Permedica	Mirai reverse	Yes		0	0	1	0	0	1	0	1	0	1		0	0	1	0	0	1	0	1	0	1

The analysis showed two predominant glenosphere size groups (e.g., 36 mm and 42 mm, see Fig. 1).

In Table 1 the availability of nickel-free implants regarding the specific prostheses is listed.

## Discussion

As the use of reverse shoulder arthroplasty continues to grow, the rate of revision is also expected to increase. Most frequently instability (38%), infection (22%), humeral (10%) and glenoidal component loosening (9%) are the main reasons for revision surgery according to Boileau et al. [2]. Zumstein et al. confirm these numbers, identifying instability and infection as the major reasons for postoperative complications [16].

Mix and Match implantation, though legally complex, is sometimes necessary, particularly during revision surgery. According to Peters et al. in this scenario the surgeon becomes the juridical manufacturer of the implant and is responsible for the functionality of the prosthesis. Therefore, mix and match should be avoided whenever possible and only used with patient informed consent [8, 13].

In the literature this is usually described for Total Knee Arthroplasty (TKA) and THA. For these joints guidelines already have been published by the European Federation of National Associations of Orthopaedics and Traumatology (EFORT) that help surgeons to make the right decision with their patient in such situations [13].

Results can also be observed regarding mix-and-match surgery of THA. Implant register studies from the Netherlands reporting a prevalence of 11% for Mix and Match in THA [9]. Tucker et al. published a study by the National Joint Registry of England and Wales (NJR) in which they found over 90,000 cases of mix-and-match THA. They found the revision rates to be lower for mixed hip prostheses, when the implanted stem and head were from the same manufacturer. In these cases, only the cup came from a different manufacturer. If the stem and head were also from different manufacturers, the revision rate was significantly higher [14].

Regarding mix and match data for rTSA remain limited, though early clinical outcomes appear encouraging [10, 11]. In a two-year follow-up there were no significant differences in the comparison groups regarding function scores [10]. Comparable revision rates are documented after a one year follow up of matched and mismatched rTSAs [11]. Also in anatomical shoulder arthroplasty, a case report describing the use of a mix-and-match technique is available. Tsolis et al. reported a case with a complication-free follow-up period of two years [12].

If the decision is made to perform a mix and match operation, it is important that the sizes of the articulating joint partners are congruent. Mattei et al. showed that size tolerances for nominally identical sizes and the associated clearance differences have a considerable influence on PE wear [6]. Since it can be assumed that different manufacturers have different manufacturing tolerances, this may contribute to increased polyethylene wear. Differences in manufacturing tolerances between companies may therefore could impact long-term outcomes.

To achieve the highest possible congruency in case of mix and match, a combination was considered potentially compatible when the diameters of the glenosphere and the humeral liner were identical.

While the variation in component sizes (see Table 1) allows for a high degree of individualized treatment, it complicates component compatibility during revision surgery. As outlined above, two predominant glenosphere size groups can be identified (see Fig. 1). Although nominal sizes may appear comparable across different manufacturers, even minor dimensional differences may preclude interchangeability.

The availability of nickel-free implant options also varies between manufacturers (see Table 1). In patients with known metal allergies, this represents an additional challenge for the surgeon when selecting appropriate components.

Standardization of rTSA component sizes, analogous to developments in Total Hip Arthroplasty, may therefore be beneficial.

This study is limited by its focus on articular component sizes. Baseplates, stems and lateralization strategies were not analyzed. The biomechanical implications of combining non-matching components remain unknown and require further research. Its recommendable restricting mix-and-match use to in‑line glenoid and humeral components. Combining stems, metaphyseal components or liners across manufacturers does not appear advisable.

Studies on the combination of materials from different manufacturers with consequently unknown coefficients of friction and potentially different manufacturing tolerances, wear or other parameters that could be used to assess actual compatibility are rare in the literature.

There is a lack of in vivo evaluations and biomechanical wear testing for mixed prosthetic components. Consequently, no definitive conclusions can be drawn as to whether specific combinations of mixed and matched prostheses are functionally compatible or safe.

The use of such combinations must therefore be considered off-label and requires thorough patient information and explicit informed consent prior to implantation. Nevertheless, it represents a pragmatic approach in select revision cases and has been described in the literature [10–12].

## Conclusion

Mix-and-match implantation is widely used and well established in THA, particularly for revision procedures. Literature regarding rTSA is rare despite increasing implantation rates. This study provides an overview of currently available prosthetic systems and potential mix-and-match combinations in reverse shoulder arthroplasty. However, the lack of biomechanical wear testing represents a significant limitation and highlights the need for further investigation.

## Data Availability

No datasets were generated or analysed during the current study.
